# Shedding New Light on the Hull-Pericarp Adhesion Mechanisms of Barley Grains by Transcriptomics Analysis of Isogenic *NUD1* and *nud1* Lines

**DOI:** 10.3390/ijms252313108

**Published:** 2024-12-06

**Authors:** Sophia V. Gerasimova, Anna M. Korotkova, Tamires de S. Rodrigues, Alexander Vikhorev, Ekaterina V. Kolosovskaya, Gennady V. Vasiliev, Michael Melzer, Christian W. Hertig, Jochen Kumlehn, Elena K. Khlestkina

**Affiliations:** 1 Institute of Cytology and Genetics of the Siberian Branch of the Russian Academy of Sciences, 630090 Novosibirsk, Russia; korotkova@bionet.nsc.ru (A.M.K.);; 2 N.I. Vavilov All-Russian Research Institute of Plant Genetic Resources (VIR), 190000 Saint Petersburg, Russia; 3 Leibniz Institute of Plant Genetics and Crop Plant Research (IPK), 06466 Gatersleben, Germany; melzer@ipk-gatersleben.de (M.M.); hertig@ipk-gatersleben.de (C.W.H.);; 4 Genomics for Climate Change Research Center (GCCRC), Universidade Estadual de Campinas, Campinas 13083-875, Brazil; tamisrod@unicamp.br; 5 Faculty of Natural Sciences, Novosibirsk State University, 630090 Novosibirsk, Russia

**Keywords:** *Hordeum vulgare*, cuticle, naked barley, hulled barley, KCS, 3-ketoacyl-CoA synthase, GDSL, cementing layer

## Abstract

In barley having adherent hulls, an irreversible connection between the pericarp with both palea and lemma is formed during grain maturation. A mutation in the *NUDUM 1* (*NUD1*) gene prevents this connection and leads to the formation of barley with non-adherent hulls. A genetic model of two isogenic lines was used to elucidate the genetic mechanisms of hull adhesion: a doubled haploid line having adherent hulls and its derivative with non-adherent hulls obtained by targeted mutagenesis of the *NUD1* gene. Comparative transcriptomics analysis of the grain coats was performed at two stages of development: the milk stage, when the hulls can still be easily detached from the pericarp, and the dough stage when the hull adhesion process occurs. It was shown that the main differences in the transcriptomes lie in the genes related to DNA replication and chromatin assembly, cell wall organization, and cuticle formation. Meanwhile, genes involved in lipid biosynthesis mostly show minor differences in expression between stages and genotypes and represent a limited set of active genes. Among the 3-ketoacyl-CoA synthase (KCS) genes active during grain development, candidates for key enzymes responsible for very long-chain fatty acid elongation were identified.

## 1. Introduction

The mechanisms resulting in the strong adherence of lemma and palea to the barley grain have been a mystery. The definition of barley grain as hulled or naked is based on its ability to be easily threshed. In the case of hulled barley, threshing does not allow the removal of the lemma and palea, while naked barley is completely cleared of the hull by threshing. The formation of naked or hulled grain is controlled by the *NUDUM 1* (*NUD1*) gene in a monogenic manner, which has been repeatedly confirmed by both forward [1] and reverse [2] genetics methods, as well as genome-wide association studies (GWAS) [3]. At the same time, NUD1 is a transcription factor and hence assumed to have target genes that have remained unknown, and there were no plant models available to reveal the mechanisms behind hulled/naked grain formation. It is commonly accepted that NUD1 belongs to the WAX INDUCER 1/SHINE 1 (WIN1/SHN1) family of transcription factors that play their roles [1] in lipid biosynthesis, particularly for the formation of the cuticle and cuticular wax on the epidermal surface [4,5,6,7]. It was suggested that the activation of a specific lipid biosynthesis process causes lipid accumulation at the pericarp surface, which entails the irreversible attachment of lemma and palea with the pericarp [1]. The closest homolog to the *NUD1* gene in barley, the *WIN1* gene, is responsible for the synthesis of diketones and related compounds during the formation of the glaucous wax coating on the generative shoots of barley. The genes regulated by the WIN1 transcription factor were identified by transcriptome analysis of isogenic lines mutated for the *WIN1* gene. It was established that the WIN1 transcription factor modulates the activity of a genetic cluster encoding enzymes in the biochemical pathway of diketone biosynthesis [8].

In this study, we took a similar approach using isogenic lines with functional *NUD1* and its knockout (KO) derivative. This pair of lines was previously obtained through targeted mutagenesis [2]. Isogenic lines generated through genome editing, with modifications in just one transcription factor gene, serve as an ideal model for identifying genes regulated by this transcription factor, as well as genes involved in associated processes.

The key distinction between hulled and naked barley lies in the formation of a lipid-based cementing layer between the pericarp at the inner side and lemma and palea outside during grain maturation in hulled barley [9]. At the milk maturity stage, this layer has not yet developed, allowing the lemma and palea to separate easily from the pericarp. In naked (hull-less) barley, this easy separation is still possible at maturity. Of note, adherent hulls can only be removed destructively [10]. In the present study, a transcriptome analysis was performed at two stages of grain maturation: at the milk maturity stage (GS73, [11]), when the lemma and palea are not yet connected with the pericarp, and at the dough maturity stage (GS83), when the cementing layer forms and the adherent hull phenotype becomes evident (Figure S1).

## 2. Results

### 2.1. Comparative Transcriptomics Reveal Genes Specifically Associated with the Formation of the Hulled Barley Grain

The libraries obtained from experimental samples cluster into four groups, corresponding to WT and *nud1* KO genotypes at the milk (MS) and dough (DS) stages of seed maturity. The difference between developmental stages is more pronounced than the difference between genotypes (Figure S2), which aligns well with expectations, as the maturation processes are similar in both lines, and the *NUD1* gene mutation does not have a pleiotropic effect.

**Figure 1:**
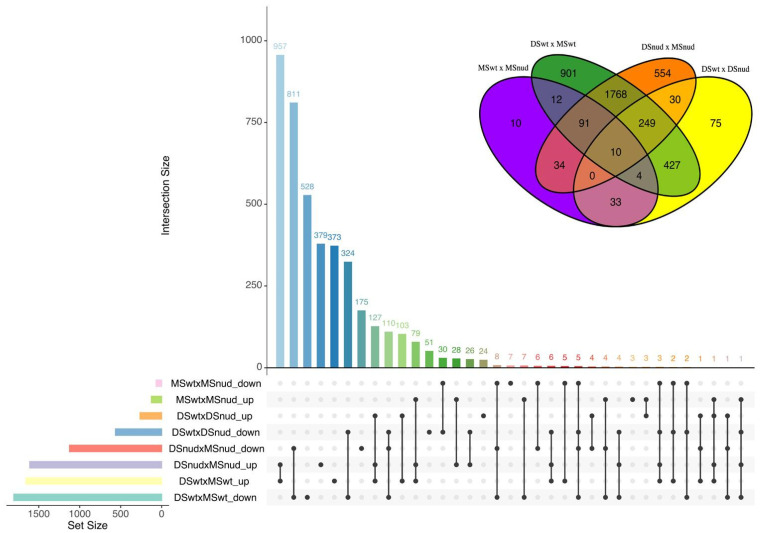
Transcriptome comparison between outer grain tissues of *nud1* knockout and wild-type lines at different developmental stages. UpSet plot shows the intersection of differentially expressed genes (DEGs) across four comparisons: MSnud vs. MSwt (milk stage, *nud1* knockout vs. wild-type), DSnud vs. DSwt (dough stage, *nud1* knockout vs. wild-type), and their respective upregulated and downregulated subsets. The *x*-axis represents the total number of DEGs in each category, and the *y*-axis indicates the number of shared DEGs between the compared variants. Venn diagram illustrates the overlap of DEGs across the four comparisons, highlighting the shared and unique gene sets. MSnud, DSnud: transcriptomic libraries from the *nud1* knockout line at the milk and dough stages, respectively. MSwt, DSwt: transcriptomic libraries from the wild-type Golden Promise line at the milk and dough stages, respectively.

All differentially expressed genes (DEGs) identified by comparing stages and genotypes are presented in Figure 1 and corresponding Tables S1–S4. At the milk stage, genotypes show comparatively few DEGs, whereas the differences increase drastically at the dough stage. The groups of DEGs identified in the milk and dough stage between the genotypes have only little overlap, suggesting that the main difference in gene expression patterns emerges only at the dough stage of grain maturity, and this is associated with certain stage-specific mechanisms. Both genotypes exert substantial changes in gene expression between the stages, with the majority of DEGs being shared, which indicates common processes of normal grain development in both hulled and naked barley. At the same time, hulled barley features a greater number of DEGs between the stages than naked barley, and most probably, this difference is associated with the development of the hull-pericarp adhesion. To define the group of the genes most specifically associated with cementing layer development, we identified the common DEGs for two lists: the comparison between WT and *nud1* KO at the dough stage (DSwtxDSnud) and the list of WT-specific DEGs from gene expression comparison between the stages (DSwtxMSwt-DSnudxMSnud). These lists share 431 DEGs, with 328 genes being down-regulated in hulled barley, and 103 genes being up-regulated. Consequently, these genes reflect a specific difference associated with the gluing of the pericarp and hull. This collection of genes was analyzed further to reveal possible genetic mechanisms of hull adhesion (Table S5). Gene ontology (GO) analysis was performed to reveal GO terms that are the most specific for up- and down-regulated genes separately. Among the genes that specifically increase expression in hulled barley, there is a significant enrichment in GO terms related to stress, tryptophan and tetrapyrrole biosynthesis, cell wall and membrane organization, and lipid metabolism (Figure S3a). On the other hand, the 328 down-regulated genes in hulled barley were mainly related to DNA replication and chromatin assembly, including those associated with DNA catabolism, checkpoints of DNA damage, DNA helicase activity, DNA-directed DNA polymerase activity, etc. Further enriched GO terms include cell wall biogenesis, cell wall modification, chitinase activity, and fatty acid and lipid biosynthesis (Figure S3b).

**Figure 2:**
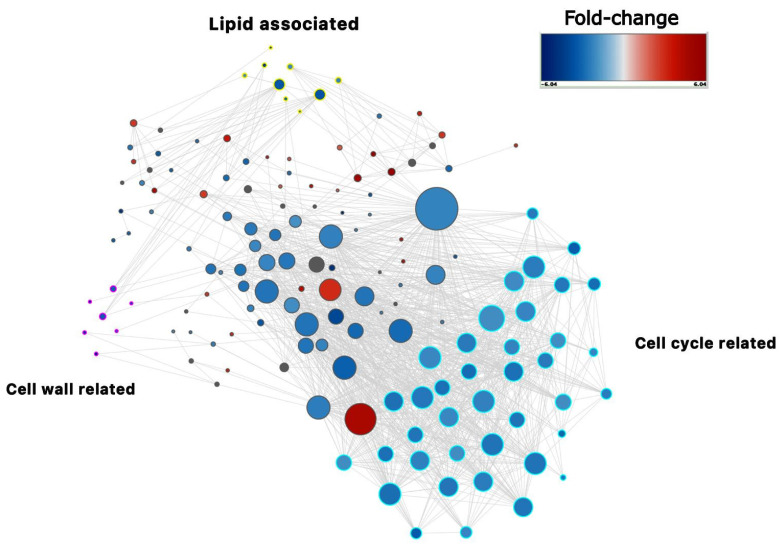
Protein-protein interaction (PPI) network building from the gene expression analysis of the 431 differentially expressed genes in hull-less barley (*nud1*) compared to WT (*NUD1*) at the dough stage. The image illustrates three main clusters related to cell cycle regulation (neon blue border), lipid metabolism (yellow border), and cell wall organization (pink border).

Notably, among the 328 genes down-regulated in hulled barley, a broad cluster of cell cycle-associated genes was identified, including 57 histone genes and 45 replication and cell division-associated genes. A protein-protein interaction (PPI) network analysis was conducted to understand better the functional relationships and interactions among these genes (Figure 2). The PPI networks, built with 189 nodes and 1261 edges in total, revealed key clusters associated with the cell cycle overall, including DNA replication, chromatin assembly, and cell cycle progression, suggesting that the inhibition of these pathways may be a coordinated response during hulled barley grain formation (Figure 2, neon blue border). The second cluster includes lipid metabolism-related genes, including lipid transfer, lipid biosynthesis, and cuticle organization genes (Figure 2, yellow border). The third cluster is formed by the cell wall-related genes (Figure 2, pink border).

### 2.2. KCS and GDSL Family Genes Are Specifically Regulated During the Formation of the Cementing Layer Between Caryopsis and Hull

**Figure 3:**
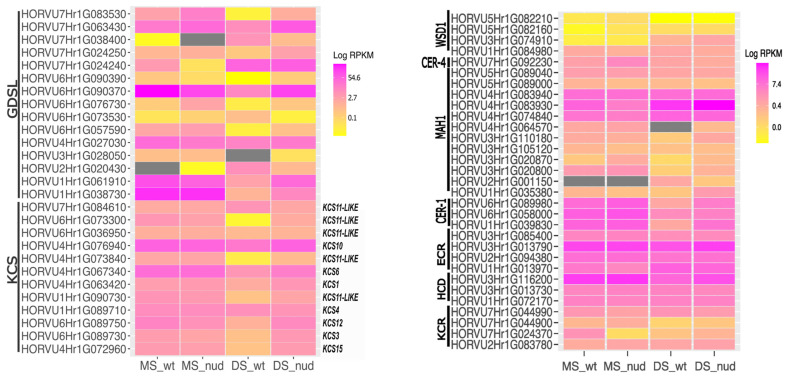
Lipid biosynthesis gene expression heatmap. Gene expression (RPKM) is shown across different developmental stages, including the milk stage (MS) and dough stage (DS), in both the wild type (wt, *NUD1*) and *nud1* knockout line (nud). KCS—3-ketoacyl-CoA synthase, KCR—3-ketoacyl-CoA reductase, HCD—b-hydroxyacyl-CoA dehydratase, ECR—enoyl-CoAreductase, CER1, CER3—acyl-CoA de-carbonylases, MAH1—midchain alkane hydroxylase, CER4—fatty acyl-CoA reductase, and WSD1—wax ester synthase. Heatmap of *Gly-Asp-Ser-Leu (GDSL) MOTIF ESTERASE/ACYLTRANSFERASE*/LIPASE (*GDSL*) and *3-KETOACYL-CoA SYNTHASE* (*KCS)* gene family expression patterns. Gene expression (RPKM) is shown across different developmental stages, including the milk stage (MS) and dough stage (DS), in both the wild-type (wt, *NUD1*) and *nud1* knockout line (nud). The gray rectangles on the heatmap indicate the absence of gene expression.

It has been shown that the normal function of the Gly-Asp-Ser-Leu (GDSL) motif esterase/acyltransferase/lipase HvGDSL1 (HORVU4Hr1G027030) is essential for the formation of the cementing layer in hulled barley [12]. This gene was identified in the list of all DEGs; it is active in all samples, but in hulled barley, a decrease in expression was observed between the milk and dough stages, whereas in naked barley, it is expressed at the same level in both stages. We decided to analyze all DEGs belonging to the GDSL family to assess their potential involvement in the adhesion process (Figure 3). Genes of this family show very diverse expression between stages and genotypes. The contrasting regulation is seen in a few GDSL family genes, including HORVU6Hr1G090370, which is homologous to the *CUTIN SYNTHASE 2* (*CUS2*) gene in *Arabidopsis thaliana* (At5g33370) [13], and the HORVU1Hr1G061910 gene being homologous to At5g45910.

A few members of the 3-ketoacyl-CoA synthase (KCS) family, responsible for elongating the very long chain fatty acids (VLCFA), are also specifically inhibited in hulled barley (Figure 3). Genes encoding KCS3, KCS5, few KCS11-LIKE, KCS12, and KCS15 are down-regulated in hulled barley at the dough stage. The *GLOSSY LEAF 1/ECERIFERUM-zh* gene (HORVU4Hr1G063420) encoding the HvKCS1 enzyme [14] and the *HvKCS6* gene (HORVU4Hr1G067340) [15] are highly active in both genotypes at both stages of development. Interestingly, genes from the KCS family exhibit three types of regulation in grain coats: some are stably expressed amongst stages and genotypes, some are down-regulated in hulled barley at the dough stage, and others are inactive in all the samples investigated (Figure 3).

### 2.3. A Limited Set of Lipid Biosynthesis Genes Is Active During Grain Maturation

The main hypothesis regarding the function of the *NUD1* gene suggests its potential influence on lipid biosynthesis genes. However, the analysis of lipid biosynthesis genes, including those involved in the fatty acid elongation (FAE) complex and the synthesis of fatty acid derivatives, did not reveal essential differences between the genotypes except for some reduction in the activity of certain lipid biosynthesis genes during the formation of the cementing layer (Figure 3). Two lipid biosynthesis genes from the *FATTY ACID HYDROXYLASE* superfamily (HORVU1Hr1G039830 and HORVU6Hr1G089980), homologous to the *CER1* gene in *A. thaliana,* are strongly inactivated in hulled barley (Table S2). In general, only a limited spectrum of lipid synthesis genes is involved in the processes occurring in the pericarp during maturation (Figure 3). Lipid biosynthesis genes exhibit strictly contrasting expression patterns: some are completely inactive, while others are active in both genotypes at both stages of development. Thus, the formation of the cementing layer is not driven by the activation of lipid biosynthesis processes; it may, in fact, rather be associated with their suppression.

## 3. Discussion

### 3.1. Formation of the Hull-Pericarp Connecting Layer Is Associated with a Specific Gene Expression Profile

The *NUD1* gene that controls the connection of pericarp and hull in barley belongs to the *WIN1/SHN1* family of transcription factors that are tightly associated with processes involving cuticle and cuticular wax formation [7,16,17]. However, it is impossible to identify either specific genes activated by this transcription factor or the specific class of compounds responsible for the formation of the cementing layer. Furthermore, the function of the *NUD1* gene does not have analogs in related cereals, as no similar adhesion mechanism between the hull and pericarp has been described for them. Overexpression of the *NUD1* gene in rice does not lead to hull-pericarp adhesion [18]. In our experiment using isogenic lines different in the functionality of the *NUD1* gene, we analyzed the transcriptomes of the pericarp at the milk stage when the connecting layer was not yet observed and at the dough stage, when adhesion should occur in hulled barley. The increase in the number of DEGs between hulled and naked barley from earlier to later stages corresponds to the dynamics of phenotype development. Hulled barley develops bigger changes in gene expression between the milk and dough stage than naked barley, suggesting the involvement of specific processes responsible for adhesion between hull and pericarp. It is likely that the *NUD1* gene’s function emerged due to a relatively recent neofunctionalization event in *Hordeum*, and it is exclusively implemented during grain maturation in the thin layer of maternal tissues at their final formation stage. The knockout of the *NUD1* gene turns off this new function, and the grain develops in a way similar to other cereals.

### 3.2. Transcription Factor NUD1 Is Rather a Repressor than an Activator

It is reasonable to consider the theory that the *NUD1* gene acts as a repressor rather than an activator when taking into account that the number of DEGs suppressed in hulled barley substantially exceeds the number of activated genes. This explains the absence of described loss-of-function mutants of key NUD1-regulated downstream genes that would exhibit a naked phenotype. The suppression of some cuticle-associated genes in hulled barley was previously reported [19], but due to the big difference between the compared cultivars, it was not possible to reveal a definite effect of the gene. In the present work, the major clusters of suppressed genes in hulled barley are associated with DNA replication and chromatin assembly, as well as cell wall formation and lipid metabolism. One can assume that differences between naked and hulled barley result from the suppression of processes of pericarp cell organization and patterning, including DNA endoreduplication, cell wall, and cuticle organization. Along with the suppression of cellular morphogenic processes, hulled barley contains markers for cell degradation, oxidative stress, and programmed cell death.

The influence of *WIN1/SHN1*-type genes on the patterns of surface-forming cells in plants has been repeatedly described [5,20]. For example, the overexpression of wheat *TaSHN1* in *A. thaliana* led to cuticle overproliferation and increased cuticle permeability, as well as changes in epidermis cell patterning [21]. In *A. thaliana*, it was shown that the function of *WIN1/SHN1* genes in floral organs is related to DELLA proteins and gibberellin biosynthesis and affects epidermal cell elongation and cuticle nano ridge formation. The silencing of the *WIN1/SHN1* genes in *A. thaliana* caused floral organs’ fusion, cutin load, cell-wall formation deficiency, and earlier abscission [6]. It is known that naked barley forms a normal cuticle, while hulled barley forms an additional cementing layer that causes adhesion between hull and pericarp, similar to organ fusion [22]. The theory that cementing layer formation is associated with increased cuticle permeability has been actively discussed in the literature [19,22,23]. Our results support this hypothesis and help identify a set of candidate genes whose inactivation may disrupt cell wall and cuticle integrity, leading to loss of barrier properties and ectopic deposition of components that make up the cementing layer. For example, the *A. thaliana CUS2* homolog (HORVU6Hr1G090370), belonging to the GDSL gene family, is suppressed during cementing layer formation. This gene is of particular interest as it is responsible for the formation of the cuticle and the presence of cuticular ridges on the mature sepal epidermis in *A. thaliana*. Mutants of this gene have increased cuticle permeability in flowers [13]. Two HOTHEAD precursor genes probably related to cutin biosynthesis [24] are also specifically suppressed at the dough stage in hulled barley. These genes might be crucial for the increase in cuticle permeability and the organ fusion-like process during hull adhesion. The regulatory genes suppressed in hulled barley included some involved in the gibberellin pathway, encoding regulators of flower and inflorescence development such as HORVU3Hr1G097810 (FLOWERING PROMOTING FACTOR-LIKE 1, [25]) and HORVU2Hr1G016630 (FRIGIDA-LIKE, [26]), and programmed cell death suppressor HORVU6Hr1G017140 (SPOTTED LEAF 11, [27]). The inactivation of the cell cycle in grain maternal tissues of hulled barley, coupled with increased oxidative stress markers, could be associated with programmed cell death activation. It has been shown that during grain maturation, DNA reduplication processes actively occur in all tissues, including the pericarp and endosperm [28]. It has also been demonstrated that the proportion of polyploid nuclei increases during early grain maturation stages and then decreases due to programmed cell death, which roughly coincides with the timing of adhesion of the pericarp with lemma and palea. It is possible that the phase of polyploid nucleus accumulation and cellular morphogenic processes last longer in naked barley, and the *NUD1* gene is required for the premature completion of the endoreduplication stage and the switch to cell degradation. The differences in *nud1* KO grains and seedlings’ resistance to abiotic stresses, in comparison to WT [29,30], especially the increased tolerance to salt stress, shown earlier [29], could be related to naked barley pericarp structure and integrity.

### 3.3. Lipid Biosynthesis Is Not Activated by the NUD1 Factor

One of the most discussed questions in the context of grain development in hulled barley is its connection to lipid biosynthesis. Lipid biosynthesis genes in barley, encoding enzymes involved in biosynthesis of fatty acids and their derivatives, exhibit considerable variability in expression across different tissues and developmental stages [8]. However, a narrowly restricted set of lipid biosynthesis genes is expressed in grain coat tissues (Figure 3). The most typical pattern for lipid biosynthesis genes was expression along all studied samples, with a decrease of expression in hulled barley at the dough stage of grain development. Studies of barley varieties with different hull adhesion [22] reported that lower proportions of alkanes and higher proportions of fatty acids and sterols were associated with stronger adhesion. The inactivation of the genes homologous to the *A. thaliana CER1* in hulled barley could be related to the reduction of alkane accumulation in the cementing layer. The *CER1* gene plays a key role in the synthesis of alkanes in the cuticular wax of both *A. thaliana* and *Brachypodium distachyon* [31,32]. The observed reduction in the activity of the *KCS* genes in hulled barley cannot explain higher FA accumulation in cultivars with firmly adherent hull and suggests a general decrease in lipid biosynthesis in hulled barley compared to its naked counterpart. It is unlikely that the *NUD1* gene activates any the lipid biosynthetic processes because no lipid biosynthesis genes are suppressed in naked barely in contrast to hulled barley.

### 3.4. New Candidate Genes for the Control of VLCFA Elongation in Barley

We hypothesized that the lipid biosynthesis genes expressed in grain coat tissues represent a “necessary and sufficient” set for the biosynthesis of major lipid compounds. The most interesting is the KCS family, which is responsible for VLCFA elongation. The elongation of the carbon chain of fatty acids occurs in the endoplasmic reticulum by the FAE complex through the sequential addition of two carbons in a condensation reaction between the elongating acyl-CoA chain and malonyl-CoA, followed by subsequent reduction reactions [33]. Each stage of elongation is catalyzed by a chain-length-specific KCS enzyme. The mechanism for VLFA synthesis is well-established in *A. thaliana*, and expression studies of different KCS genes in yeast have allowed for a precise investigation of each enzyme’s specificity and the genetic control of each stage of fatty acid elongation [34]. Barley KCS genes have not yet been fully characterized. In total, 33 candidate genes containing the KCS domain were identified in barley [35]. Only two KCS enzymes in barley have been characterized in terms of their chain length specificity. The *GLOSSY LEAF 1/ECERIFERUM-zh* gene (HORVU4Hr1G063420) encoding the HvKCS1 enzyme is specific for acyl chain lengths between 16 and 20 carbons [14]. This gene is homologous to *A. thaliana KCS1*, which is also responsible for the production of saturated and monounsaturated C20 and C22 fatty acids. The barley *HvKCS6* gene (HORVU4Hr1G067340) is responsible for VLCFA elongation from C24 to C26 and the formation of corresponding C26 alcohols [15]. It is homologous to both *Arabidopsis KCS5* and *KCS6*, which produce C24 to C28 VLCFA. Genes responsible for elongation between 20 and 24 carbons and 26-32 carbons are not yet characterized in barley.

Previous research has assessed the expression of KCS family genes in barley across different tissues [8,35]. Six of the genes that showed stable expression in this study were also highly stable in previous works. These genes include *HvKCS1* and *HvKCS6*, and four genes encoding enzymes that are not yet characterized, namely *HvKCS4* (HORVU1Hr1G089710), *HvKCS10* (HORVU4Hr1G076940), and two *HvKCS11*-*LIKE* (HORVU7Hr1G084610, HORVU6Hr1G036950). These four genes are candidates for the missing components of the FAE complex in barley (Table 1). *HvKCS11*-*LIKE* genes are homologous to *AtKCS11*, which is responsible for the synthesis of C20, C22, C24, and C26 fatty acids. *HvKCS10* is homologous to *AtKCS10*, and *AtKCS15* promotes the accumulation of C22-C24 and C22-C26 VLCFA. *HvKCS4* is homologous to *AtKCS17* and *AtKCS4*, which are responsible for VLCFA chain lengths between C24 and C28.

The identified genes likely fill gaps in our understanding of the stages of fatty acid biosynthesis in barley. A presumable model for the whole-length FAE-related VLCFA elongation in barley is the following: HvKCS1 is the enzyme catalyzing the initial stages of elongation; it extends the molecule to C20. Subsequently, HvKCS10 and HvKCS11 primarily catalyze two additional cycles of elongation, forming C22 and C24 VLCFA, respectively. HvKCS6 is responsible for forming C26 VLCFA, and HvKCS4 contributes to the formation of C28 molecules. Further elongation involves the simultaneous activity of KCS and CER2-LIKE enzymes [36].

## 4. Materials and Methods

### 4.1. Plant Material

Two-rowed spring barley (*Hordeum vulgare* L.) isogenic lines 22-1 (WT) and 05-4 (with 1 nt insertion in the *NUD* gene) with the cv. Golden Promise background was used for transcriptome analysis. Until the generative stage, the plants were grown at 14 °C with a 12-h photoperiod. Then, growth continued under conditions of 17 °C and a 16-h photoperiod. Grains were collected at early milk (GS73) and early dough (GS83) stages of development [11]. The lemma, palea, starchy endosperm, and embryo were manually removed, and the rest of the grains were collected for RNA extraction. The experiment was performed in four biological replicates; each replicate consisted of a pool of three plants, with two grains collected from each plant.

### 4.2. RNA-Seq

RNA extraction was performed using the RNeasy Micro Kit (QIAGEN, Venlo, Netherlands) according to the manufacturer’s protocol. Library preparation for sequencing was conducted using the TruSeq^®^ Stranded mRNA LT Sample Prep Kit (Illumina, San Diego, CA, USA). Sequencing was carried out using the Illumina NextSeq 550 (San Diego, CA, USA). Quality assessment of the raw reads was performed using FASTQC v. 0.11.9. Quality filtering of the libraries was done using the fastp program v. 0.23.4. Filtered reads were then mapped to the barley genome assembly IBSC v.2 release 47, obtained from the EnsemblPlants database (https://plants.ensembl.org/Hordeum_vulgare/Info/Index accessed on 15 January 2022). The DART program was used for genome reference mapping. The mapped reads were quantified using the featureCounts function from the Subread package v. 2.0.4. Principal component analysis (PCA) was performed using the plotPCA function from the DESeq2 package version 1.28.1 [37].

A total of 16 RNA libraries were sequenced, yielding 491.69 million reads, each 75 base pairs long. On average, each sequenced library contained 30.73 million reads, with an average nucleotide quality above Q20 = 96.24%. After filtering with the fastp program, 476.50 million reads (96.91%) remained, and the proportion of nucleotides with Q20 quality increased to 97.16%. On average, 90.11% of the reads were mapped to the reference genome, of which 70.67% were uniquely mapped. A total of 21,117 genes with significant levels of expression were detected.

Principal component analysis (PCA) showed that libraries from the same condition clustered together, indicating the high quality of the libraries. The exception was the MS_NUD3 library, which fell outside the cluster and was excluded from further analysis, resulting in three biological replicates for the *nud1* KO line at the milk stage. Differential expression analysis was performed using the edgeR package v. 3.30.3 in R. Generalized linear models (GLM) were used to search for DEGs. Genes with FDR (adjusted *p*-value for multiple testing) < 0.05 and logFC (logarithm of fold change) > 2 were considered significantly differentially expressed. Annotation of DEGs was conducted using AgriGO v2 and ReviGO tools. Gene ontology terms with FDR < 0.05 were considered significantly enriched. Genes related to wax biosynthesis were identified using the KEGG database. Co-expressed genes were identified using k-means clustering. Enrichment analysis of transcription factor binding sites in differentially expressed and co-expressed genes was performed using the iDEP96 tool. The DEGs were processed using the BioMart tool in Phytozome v.13 to convert barley gene IDs into their corresponding UniProt IDs. These UniProt IDs were subsequently used as input in the STRING database to obtain the protein-protein interaction (PPI) network for *Hordeum vulgare*. The network was built using Cytoscape v.3.7.2, and clusters corresponding to enriched GO terms were highlighted for further analysis. The obtained quality of RNA-seq was considered to be robust based on three to four biological replicates with a low deviation of corresponding datasets and a high rate of uniquely mapped reads. According to the last evidence that RNA-seq results with sufficient quality do not require qPCR validation [38], it was decided to restrict the qPCR validation experiment to a few random genes with different expression patterns. Five genes were selected from the DEG lists, and the nucleotide sequences of the genes were obtained from the BARLEX database (https://apex.ipk-gatersleben.de/apex/f?p=284:10, accessed on 30 January 2022). Primers were designed to target the coding regions of these genes (Table S5). The *UBIQUITIN* gene was used as a reference. qPCR analysis was conducted with three biological and three technical replicates for each sample. The experiments were performed using the AccuSEQ Real-Time PCR Software v. 3.2. The BioMaster HS-qPCR Lo-ROX SYBR kit (Biolabmix, Novosibirsk, Russia) was used for the qPCR. The expression patterns obtained by qPCR and RNAseq proved to be similar (Figure S4).

### 4.3. Light Microscope

Grains of *Hordeum vulgare* WT and *nud1* were used for microwave-assisted aldehyde fixation, substitution, and embedding as described (Table S6). Semi-microtomy, Methylene Blue/Azur II staining, and histological analyses were performed as previously described [39].

## 5. Conclusions

The hull/naked grain trait in barley is a key agricultural characteristic, influencing grain processing costs, dietary value, and suitability for various end uses. Transcriptomic analysis has revealed distinct gene expression profiles during the maturation of hull-less *nud1* KO and hulled WT grains, highlighting significant differences in processes such as DNA replication, cell wall organization, cuticle formation, and lipid transport. These findings are particularly intriguing, as they suggest that the NUD1 transcription factor plays a regulatory role in cellular processes within the pericarp. Furthermore, they indicate that pericarp cells in hull-less barley may be more metabolically active, potentially contributing to its enhanced dietary value. The results of this study also challenge the hypothesis that NUD1 specifically activates lipid biosynthesis. Instead, NUD1 may act as an inhibitor of certain cellular processes and a promoter of cellular degradation, leading to increased permeability of the cell walls and cuticle in hulled barley. Additionally, a limited set of active lipid biosynthesis genes was identified in the barley pericarp, allowing us to pinpoint key candidate genes responsible for different steps of fatty acid elongation in barley.

## Figures and Tables

**Table 1:** Co-expressed set of barley KCS genes and their potential role in fatty acid elongation.

Gene	ID	*A. thaliana* Homolog	Fatty Acid Length
*HvKCS1*	HORVU4Hr1G063420	AT1G01120	C16-C20
*HvKCS10*	HORVU4Hr1G076940	AT2G26250	C22-C24
*HvKCS11-LIKE*	HORVU7Hr1G084610, HORVU6Hr1G036950	AT2G26640	C22-C24
*HvKCS6*	HORVU4Hr1G067340	AT1G68530	C24-C26
*HvKCS4*	HORVU1Hr1G089710	AT1G19440	C28

## Data Availability

The original data presented in the study are openly available in the NCBI Sequence Read Archive (SRA) at https://www.ncbi.nlm.nih.gov/sra/PRJNA1186979 (accessed on 18 November 2024).

## Supplementary Materials

The following supporting information can be downloaded at: https://www.mdpi.com/article/10.3390/ijms252313108/s1.
