# Supplementary material for: Shedding New Light on the Hull-Pericarp Adhesion Mechanisms of Barley Grains by Transcriptomics Analysis of Isogenic NUD1 and nud1 Lines
Source: Int J Mol Sci. 2024 Dec 6;25(23):13108. doi: 10.3390/ijms252313108 (PMC11641734; doi:10.3390/ijms252313108)
Supplement: Supplementary file 1 [file ijms-25-13108-s001.zip › Gerasimova_IJMS_supplementary_R1.pdf]

Shedding New Light on the Hull-Pericarp Adhesion Mechanisms of Barley Grains by Transcriptomics Analysis of Isogenic *NUD1* and *nud1* Lines

Sophia V. Gerasimova, Anna M. Korotkova, Tamires de S. Rodrigues, Alexander Vikhorev, Ekaterina V. Kolosovskaya, Gennady V. Vasiliev, Michael Melzer, Christian W. Hertig, Jochen Kumlehn, Elena K. Khlestkina

Supplementary Figures

Figure S1. Plant material representative for the RNA-seq experiment. (a) Spikes, spikelets and grains of cv. Golden Promise *NUD1* (wild-type, WT) and *nud1* knockout isogenic lines across milk and dough developmental stages of grains. (b) Light microscopy images of tissues taken for RNA extraction at the dough stage of grain development. Black bars at the right side indicate the sampled tissue layers. MS_wt, MS_nud – wild-type Golden Promise line and *nud1* knockout line respectively at the milk stage of grain development; DS_wt, DS_nud - wild-type Golden Promise line and *nud1* knockout line respectively at the dough stage of grain development;

(a)

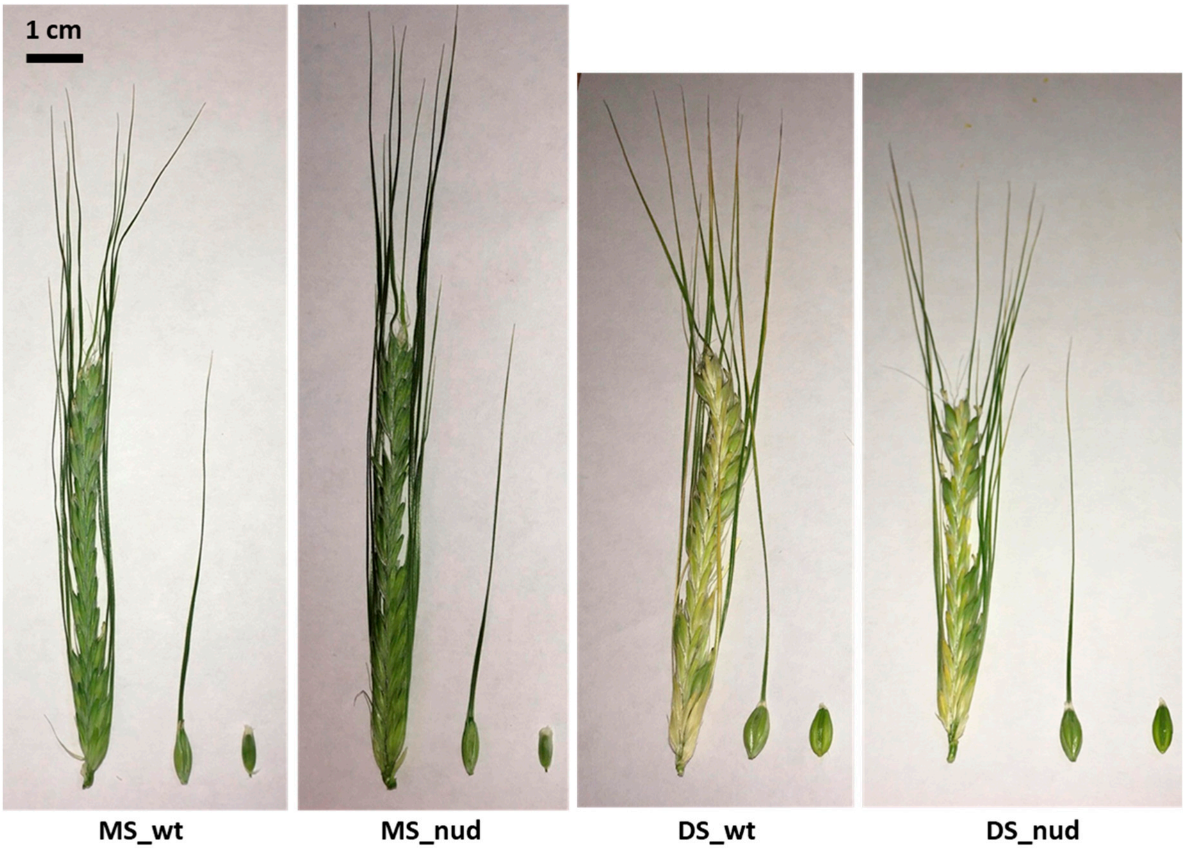
1 cm
MS_wt
MS_nud
DS_wt
DS_nud

(b)

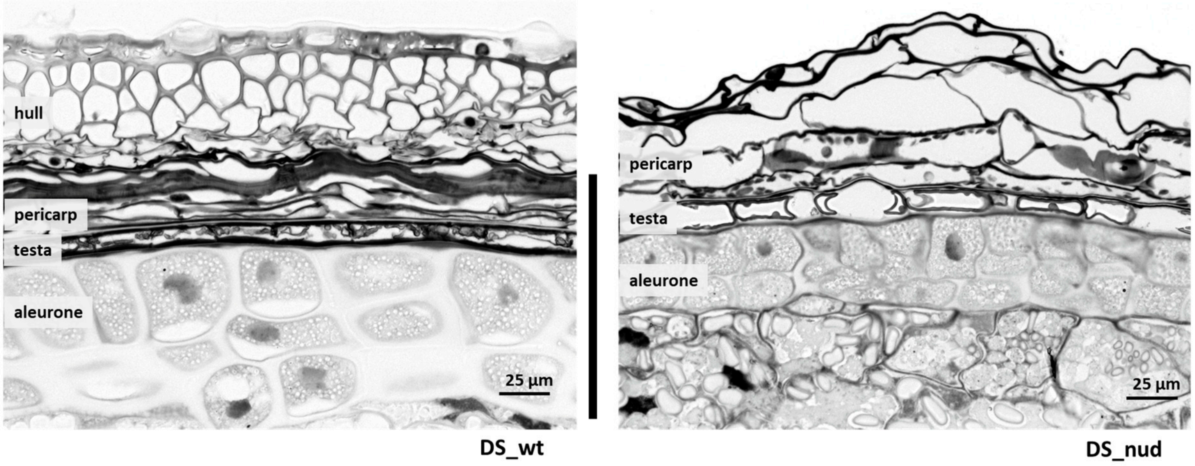
hull
pericarp
testa
aleurone
25 µm
DS_wt
pericarp
testa
aleurone
25 µm
DS_nud

Figure S2. Principle components analysis (PCA) of transcriptome libraries. MSnud, DSnud: transcriptomic libraries from the nud1 knockout line at the milk and dough stages, respectively. MSwt, DSwt: transcriptomic libraries from the wild-type Golden Promise line at the milk and dough stages, respectively.

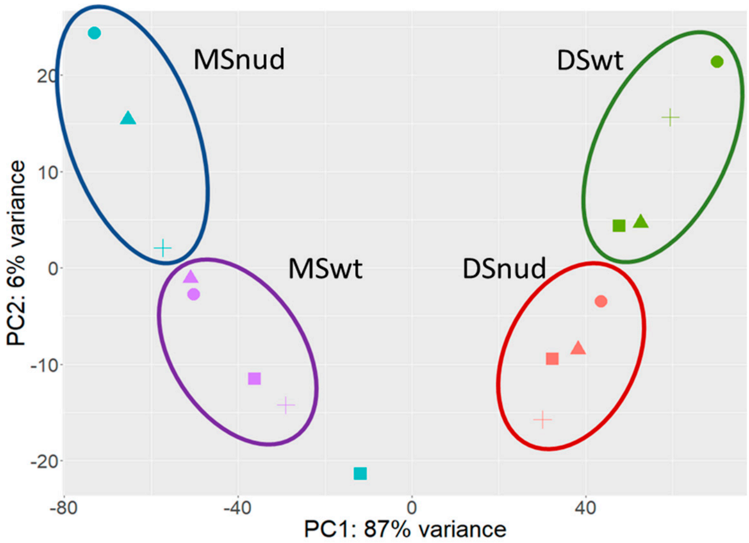

Figure S3. Gene Ontology (GO) enrichment analysis of the 431 differentially expressed genes in hulled barley compared to WT at the dough stage. (a) Up-regulated genes. (b) Down-regulated genes. GO terms semantic analysis was performed on ReviGO tool. Frequency represents the number of times a GO term is associated with a semantic group, while log size corresponds to the total GO annotations related to those categories.

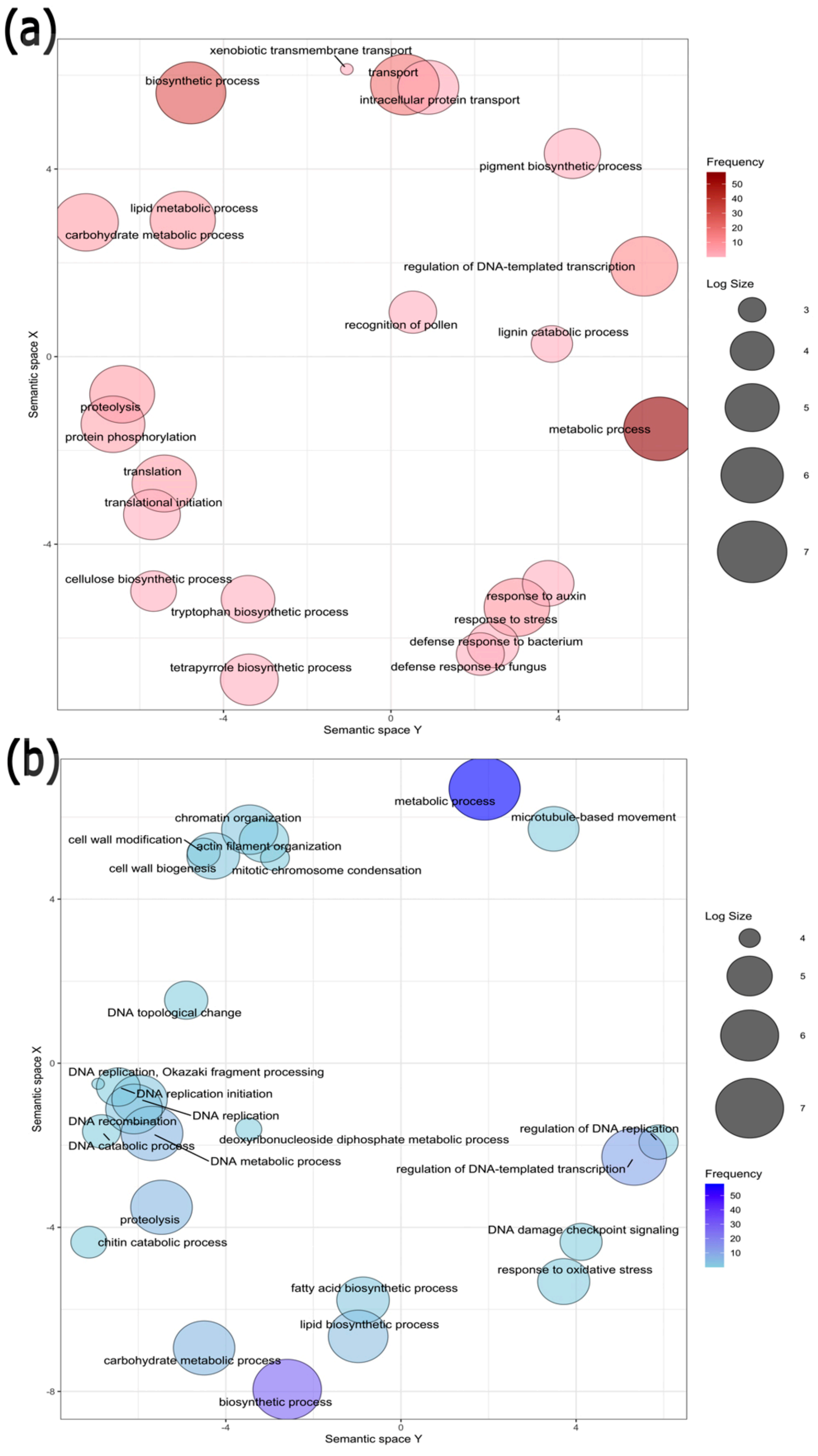
(a)
xenobiotic transmembrane transport
biosynthetic process
transport
intracellular protein transport
pigment biosynthetic process
lipid metabolic process
carbohydrate metabolic process
regulation of DNA-templated transcription
recognition of pollen
lignin catabolic process
proteolysis
protein phosphorylation
metabolic process
translation
translational initiation
cellulose biosynthetic process
tryptophan biosynthetic process
response to auxin
response to stress
defense response to bacterium
defense response to fungus
tetrapyrrole biosynthetic process
Semantic space X
Semantic space Y
Frequency
50
40
30
20
10
Log Size
3
4
5
6
7
(b)
metabolic process
chromatin organization
microtubule-based movement
cell wall modification
actin filament organization
cell wall biogenesis
mitotic chromosome condensation
DNA topological change
DNA replication, Okazaki fragment processing
DNA replication initiation
DNA replication
DNA recombination
deoxyribonucleoside diphosphate metabolic process
regulation of DNA replication
DNA catabolic process
DNA metabolic process
regulation of DNA-templated transcription
proteolysis
chitin catabolic process
DNA damage checkpoint signaling
response to oxidative stress
fatty acid biosynthetic process
lipid biosynthetic process
carbohydrate metabolic process
biosynthetic process
Semantic space X
Semantic space Y
Log Size
4
5
6
7
Frequency
50
40
30
20
10

Figure S4. RT-qPCR validation of the RNAseq dataset. Five genes were selected from the transcriptome analysis for validation (n=3). Gene expression levels from RNAseq (RPKM values) and qPCR (relative expression) are displayed across different developmental stages: milk stage (MS) and dough stage (DS), in both the wild type (WT) and the *nud1* knockout line (nud). RNAseq and qPCR data were normalized using the MS_WT condition expression level as baseline. The graph represents the relative expression level in log10 scale for both RNAseq (spheres) and qPCR (bars).

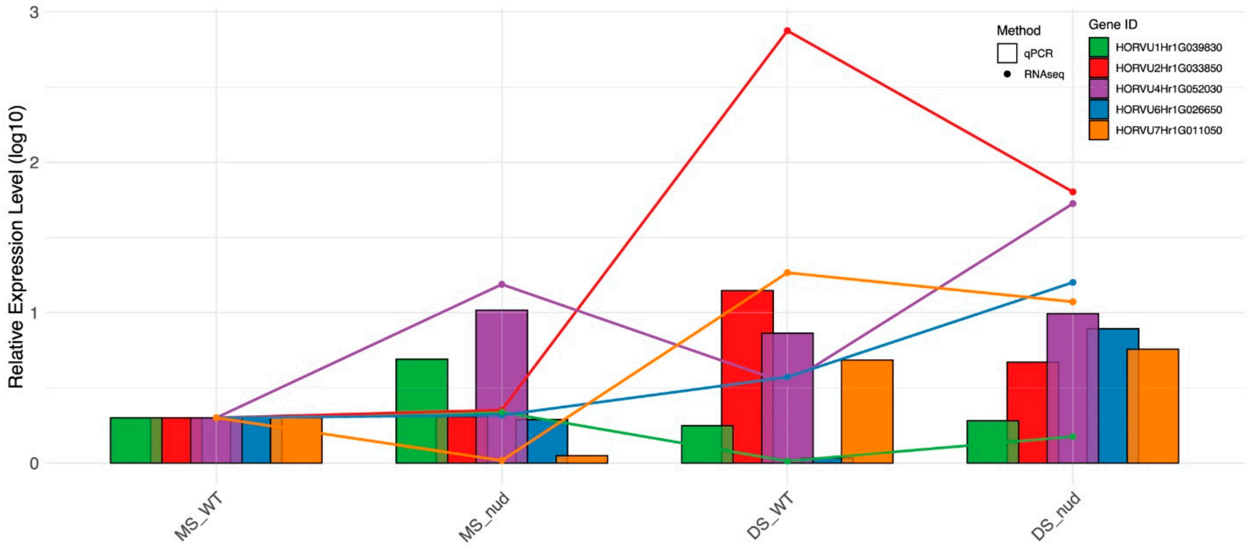

Supplementary tables

Supplementary tables S1-S4 are provided in separate files

Table S1. RPKM values obtained from RNAseq libraries of outer grain coats at different developmental stages. MS - milk stage, DS - dough stage, wt - wild type, nud - *nud1* knockout line.

Table S2. Full annotation of differentially expressed genes (DEGs) comparison between *nud* knockout and wild-type lines at different developmental stages and DEGs identified across multiple comparisons between developmental stages (milk stage and dough stage) and genotypes (WT and KO *nud*). The table includes log2FC, FDR, best rice homologs, functional annotations (GO, KO, KOG, Panther, Pfam), PPI network degree of connection, and associated clusters.

Table S3. Dataset for Venn diagram of shared genes identified across multiple comparisons between developmental stages (milk stage and dough stage) and genotypes (WT and KO *nud*). MS - milk stage, DS - dough stage, wt - wild type, nud - *nud1* knockout line.

Table S4. Dataset for upsetplot diagram of intersections identified across multiple comparisons between developmental stages (milk stage and dough stage) and genotypes (WT and KO *nud*) and their respective gene IDs. MS - milk stage, DS - dough stage, wt - wild type, nud - *nud1* knockout line.

Table S5. Primers used for qPCR

Gene	Name	Sequence	Product length
HORVU2Hr1G033850	3850_F<br>3850_R	TTACTATGCCGGAGATCCCGA<br>GGCATGGTGTGGCATTTCAG	164
HORVU6Hr1G026650	6650_F<br>6650_R	AAACTACTCTACTGGGAACAC<br>CGAGATCAATAAGGAAATCTGCTG	157
HORVU1Hr1G039830	9830-F1<br>9830-R1	ACGGTCTATTGGTCTTGTGC<br>TGCATCCGTCAAGTACCACA	70
HORVU4Hr1G052030	2030-F1<br>2030-R1	GCAGACATGACGACCACTCA<br>GGCAAAGTACTGGGCTGGTA	178
HORVU7Hr1G011050	1050-F1<br>1050-R1	CAAAAGGGTGCCGCTACAAC<br>ATGATAGGCCTGACGATGCG	100
Ubiquitin	Ubc1<br>Ubc2	ATTTGTGAAGACCCTCACCG<br>CACCAAGTGAAGGGTGGACT	201

Table S6: Protocol for combined conventional and microwave-proceeded fixation, dehydration and resin embedding of *Hordeum vulgare* grains for histological analysis.

Micowave processing in a PELCO Bio Wave®34700-230 (Ted Pella, Inc., Redding CA, USA)				
**Process**	**Reagent**	**Power [W]**	**Time [sec]**	**Vacuum [mm Hg]**
**1. Aldehyde fixation**	2.0% (v/v) glutaraldehyde and 2.0% (v/v) paraformaldehyde in 0.05 M cacodylate buffer (pH 7.3)	150<br>0<br>150<br>0<br>150	60<br>60<br>60<br>60<br>60	10<br>0<br>10<br>0<br>10
		additional 48 hrs at 4°C		
**2. Wash**	1x 0.05 M cacodylate buffer (pH 7.3) and 2x aqua dest.	150	60	0
**5. Dehydration**	Acetone series: 30%, 40%, 50%, 60%, 70%, 80%, 90%, 2x 100%	150	60	0
	for each step	additional 15 minutes on shaker		
**6. Resin infiltration**	Spurr´s resin on shaker	25% resin over night<br>50% resin 24 hrs<br>75% resin 24 hrs<br>100% resin over night		
**7. Polymerisation**	24 hrs at 70°C in beem capsules in a heating cabinet.			
